# Thermo-responsive injectable naringin-loaded hydrogel polymerised sodium alginate/bioglass delivery for articular cartilage

**DOI:** 10.1080/10717544.2021.1938752

**Published:** 2021-06-26

**Authors:** Xiang Li, Yang Lu, Yuxin Wang, Shengji Zhou, Liangping Li, Fengchao Zhao

**Affiliations:** a Department of Orthopaedic Surgery, The First Affiliated Hospital, Zhejiang University, School of Medicine, Hangzhou, China; b Department of Surgery, The Second Affiliated Hospital of Zhejiang University, School of Medicine, Hangzhou, China

**Keywords:** Articular cartilage, Naringin, injectable hydrogels, inflammatory response

## Abstract

In the human body, joint cartilage is of great importance. It has long been a big therapeutic problem to fix joint cartilage lesions as it appears due to different conditions. Recent stories have shown that the cartilage replacement process must delay the extracellular (ECM) cartilage deterioration and modulate the host's inflammation response. For the reconstruction of the articular cartilage, drug-loaded injectable hydrogels were developed. This hydrogel could retain the chondrocyte phenotype, but the host's inflammatory reaction could also be controlled. The bioglass (BG)/sodium alginate (SA) injectable hydrogels was combined with agarose (AG)/Naringin hydrogel in injectable thermal response for articular cartilage regeneration with a non-chargeable hydrogel that contains both Naringin and BG (Naringin–BG hydrogels). The Naringin–BG hydrogel has an adequate swelling ratio that encourages the fusion of tissue formed with host tissue and enables the gradual release of Naringin bioavailabilities enhanced *in situ*. The Naringin–BG hydrogel can upgrade the typical chondrocyte phenotype by upregulating aggrecan, SRY-box 9, and collagen type II alpha one chain. It may also stimulate the polarization of M2 macrophage, lower inflammations, and prevent ECM degradations through the decrease of the expressions of the indictable metalloproteinase-13 matrix, nitric oxide synthase, and metalloproteinase-1 matrix. The formed tissues were identical to normal tissues and firmly incorporated with the surrounding tissue after administering the Naringin–BG hydrogels into the rat model articular cartilage defects. Then the injectable Naringin–BG hydrogel increases the bioavailable content of Naringin and retains the chondrocyte phenotype.

## 1. Introduction

Alleviating cartilage defects was, for scientists and doctors, often a challenging task. In the subchondral bone zone, joint–cartilage defects frequently happen. The joint cartilage is a form of hyaline cartilage that shows good elastic properties, low friction and hypertonicity and plays an essential role in maintaining collective motion (Vázquez-Portalatín et al., 2016; Ye et al., 2016; Li et al., 2020). Joint replacements are not suitable for younger and medium-aged people because of inadequate life expectancy in joints implantation. Often sport-related trauma and arthritis, birth defects, discomfort or exhaustion, and other causes are caused by articular cartilage defects. With indigenous cartilage modules in the range of 300–800 kPa, mechanical efficiency and structural stability are essential requirements of cartilage scaffold manufacturing (Li et al., 2017; Chen et al., 2020; Yu et al., 2020). Mainly because there are no vascular channels, the conversion of stable chondrocytes into deficient locations and low protein efficiency typical of the adjacent extracellular matrices are inadequate. The combined administration to cartilage defect site of suitable cells, scaffolds and biological factors will contribute toward the structural and functional restoration of damaged tissues (Reimann et al., 2017; Kilmer et al., 2020; Mancipe Castro et al., 2020). Therefore, the challenge in orthopedics lies in an appropriate procedure for the reconstruction of joint cartilage defects. Recent repair methods of cartilage, including perichondral, scaphoid fracture, allogenic, and perichondral allogeneic grafts, do not offer complete and long-lasting healing (Toh et al., 2011; Lourido et al., 2014; Liu et al., 2017). Therefore, cartilage regeneration remains an obstacle in tissue engineering, though working on potential artificial cartilage replacements as regeneration strategies continue. The examination of hydrogels as effective treatment materials to substitute or regenerate weakened articular cartilage resulted in an increased emphasis on synthetic polymers. The use of hydrogels in a wide variety of fields results in their physical properties being manipulated. Hydrogels are cross-linked, biocompatible, and do not irritate the cartilage tissue when in touch. The use of biomaterials for cartilage regeneration has significantly improved cartilage regeneration in recent years (Li et al., 2014; Karimi et al., 2015; Camarero-Espinosa et al., 2016; Liu et al., 2021).

Inflammation, which is also essential in cartilage defects, is amongst the most critical variables in OA. Inflammation cytokines, such as IL-1β, IL-6 and tumour-necrosis factors α are quickly increased following cartilage damage and decomposite features such as membrane MMP-13, and metalloproteinase-1 those causes inflammations, chondrocyte cell death and cartilage deteriorations of the ECM (Eleswarapu & Athanasiou, 2013; Steele et al., 2014; Li et al., 2014; Castilho et al., 2019). Anti-inflammatory action has been shown to encourage chondrocytes to survive and to reduce the occurrence of OA. The traditional solution to suppress inflammation may be anti-inflammatory medicines. Previously engineered biomaterials with anti-inflammatory properties (Bell et al., 2014; Wahlquist et al., 2017; Yin et al., 2018). Zhou et al. constructed an inherent cartilage regeneration scaffold of silk fibroin–chondroitin, which could be enhanced by the immuno-inhibition ability of chondsulfate (CS). This prompted us to examine whether an anti-inflammatory factor that is stronger than CS could be used to identify areas of improvement (Armiento et al., 2018; Brown et al., 2019; Antich et al., 2020; Weizel et al., 2020).

Naringin is a flavonoid substance that has a high antioxidant and anti-inflammatory activity in vegetables and fruits. It can reduce the risk of heart oxidative stress diseases such as rheumatoid arthritis and osteoarthritis (Lee et al., 1999; Cao et al., 2018; Tang et al., 2018; Zhu et al., 2019; Liu et al., 2020). *In vivo* and *in vitro* macrophages are also confirmed to decrease the transcription and cytokines substantially, trigger macrophage into an M2 phenotype, and reduce the expressions of the MMP-13 (Dai et al., 2011; Singh et al., 2016; Luo et al., 2019). In preserving cartilage, Naringin has a possible therapeutic benefit. Any trials, however, did not demonstrate any evident changes in Naringin arthritis. Naringin's poor bioavailability may cause this discrepancy by oral administration. After elevated levels of Naringin orally, no free naringin might be found in human plasma (Feng et al., 2017; Jabbari et al., 2017; Heidary Moghaddam et al., 2020). Naringin also becomes a functionalized form in the human body's atmosphere. The metabolite of Naringin has a lower antioxidants potential than Naringin, which shows that Naringin's other bioefficacy could be decreased. The improvement of Naringin bioavailability is important for its therapeutic potential (Syed et al., 2020; Zarate-Vilet et al., 2020; Zhang et al., 2020).

Research in tissue-engineered fields has discovered that specific nanomaterials can control host inflammations by adding anti-inflammatory medications for intense bioactive glass (BG) (Cerruti et al., 2004; O’Donnell et al., 2010; Liang et al., 2021). We observed that BG could activate macrophage for the M2, which indicates BGs anti-inflammatory property. BG will improve wound healing in various ways, as per our previous studies. BG can also assist chondrocyte development, and the chondrogenic molecular markers stimulate (Balestriere et al., 2020; Deshmukh et al., 2020; Dai et al., 2020). Recent research was performed to improve osteochondral regeneration through an injectable BG hydrogel that contains stem cells from the bone marrow (Manissorn et al., 2021).

Provided the anti-inflammatory and cartilage protective characteristics of BG and Naringin, we assumed that Naringin and BG could simultaneously take advantage of the bioactivity of biomolecules and biomass to accelerate the treatment of cartilage defects. We also developed a hybrid composite hydrogel with Naringin to deliver a drug's mechanism to prove our hypothesis. This system has several benefits in our assumption. First of all, the clinical delivery system is injectable and is suitable for lab and therapeutic use. In addition, the pharmaceutical composite hydrogel enhances Naringin bioavailability by transferring it from orally to specific lesion action. Simultaneously, the gradual decay of the hydrogel will act as Naringin continuous release. Since cartilage repair materials' anti-inflammatory effects are being taken seriously, the combination Naringin and BG will improve the anti-inflammatory effect of the material. Furthermore, there will be an increased capacity to preserve the chondrocyte phenotype. This immunoregulatory mechanism may also have a more significant repair effect on cartilage. This injectable formulation was initially synthesized in biodegradability and release kinetics *in vitro* for the Naringin–BG hydrogel. In this study, In a rat model of osteochondral defect, the therapeutic effectiveness of the injectable formula was then examined.

## 2. Methods and materials

### 2.1. Chondrocytes culture and murine-derived macrophage cells

The cartilage tissue was divided and crushed into minor segments. The sections were first processed for 45 min in EDTA–trypsinsation solutions (0.02% EDTA and 0.25% trypsin) and then processed for 5 h in collagenase II (0.5%) and incubate shaker with 37 °C. The cells were cultured in Dulbecco Modified Eagle’s medium (DMEM) (Biosciences, China) supplemented with 1% penicillin–streptomycin and 10% fetal bovine serum (FBS) (Gibco BRL, Rockville, MD, USA) under an atmosphere of 5% CO_2_ in a humidified 37 °C incubator.

RAW 264.7 (murine-derived macrophage cells) macrophage cells were purchased from American Type Culture Collection (Manassas, VA, USA). The cells were cultured in Dulbecco Modified Eagle’s medium (DMEM) (Welgene, Korea) supplemented with 1% penicillin–streptomycin and 10% fetal bovine serum (FBS) (Gibco BRL, Rockville, MD, USA) under an atmosphere of 5% CO_2_ in a humidified 37 °C incubator (Subarkhan & Ramesh, 2016; Mohamed Subarkhan et al., 2019; Sathiya Kamatchi et al., 2020).

### 2.2. Cytocompatibility test for naringin

The Cell Counting Kit-8 test was performed to assess the biocompatibility and to determine the concentration of Naringin to rat chondrocytes. Rate chondrocytes with a density of 1 × 10^4^ cells per wine were placed on a 48-well plate and cultivated with 10% fetal bovine serum (FBS) in the DMEM-F12. RAW 264.7 cells were then treatment with Naringin (μM concentration of 5, 10 and 20, respectively) at varying doses and the cultured media was controlled. CCK-8 was employed for detecting at 12, 24, 48 and 72 h after 3 days of continuous monitoring. A microplate reader (Bio-Rad 680; Bio-Rad Laboratories Inc., Hercules, CA, United States) assessed the absorption wavelength at 475 nm.

### 2.3. Naringin–BG hydrogel fabrication

Sodium alginate was dissolved at a concentration of 2% in brown algae. The Shanghai Ceramics Institute, Chinese Academy of Science provided Bioglass (BG) powder. For sterilizing the SA solution, a 0.23-μm Millipore filter was employed. The solution of the SA was kept in 4 °C after sterilization, whereas BG powders were ultraviolet light sterilized. AG was diluted and boiled in order to make the 2% (w/v) agarose solution.

**Figure 1.**
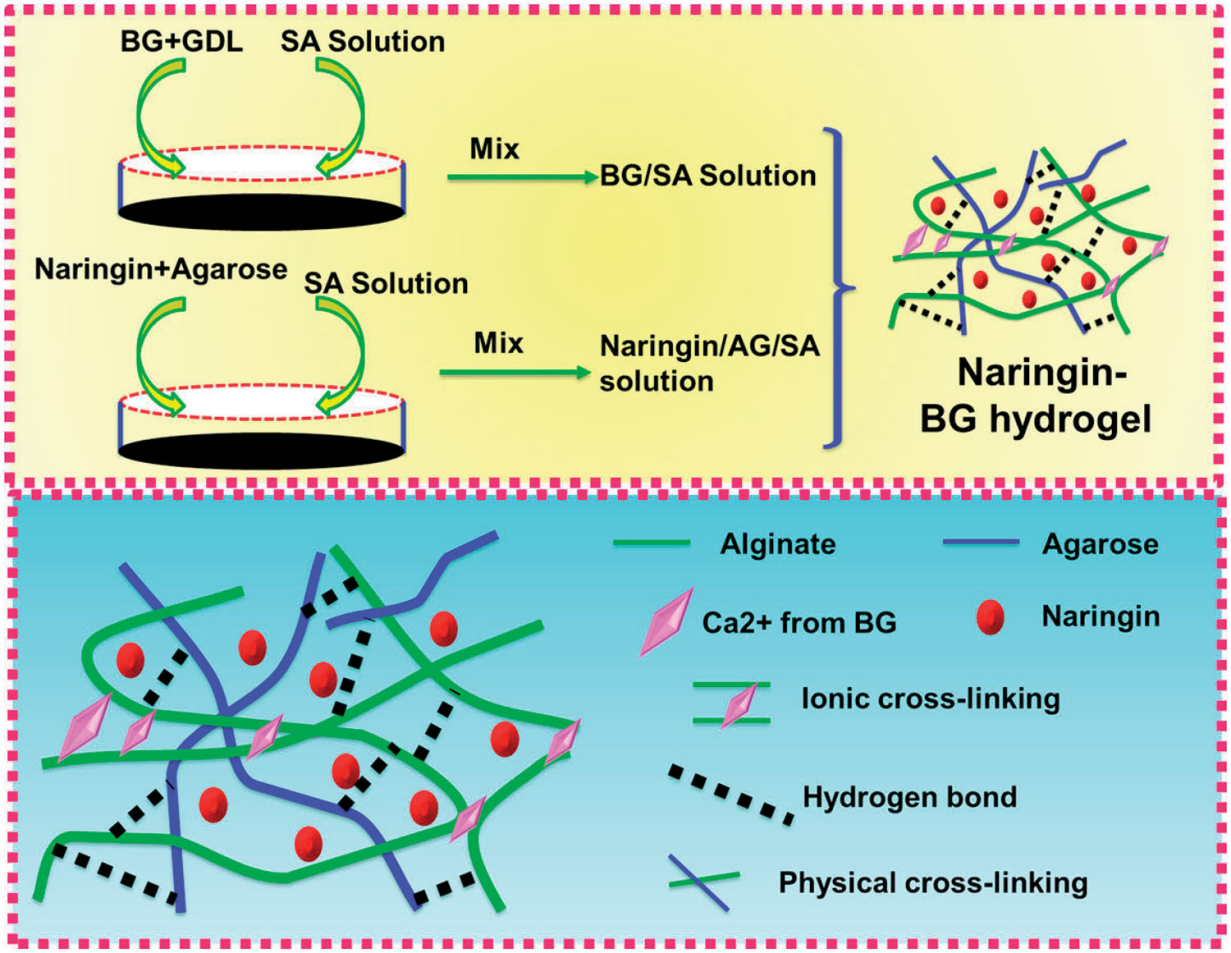
Fabrication of the Naringin–BG hydrogel model. Schematic illustration of the Naringin–BG hydrogel formation mechanism for the effective articular cartilage defects model.

Then, added in one syringe were 0.1 g of gluconolactone and 5 ml of solution of sterilized SA. In order to achieve SA/BG hydrogel 0.1 g of BG sample was weighed. 5 ml agarose solutions were added to assure the concentration of the drug loading and then combined with a 5 ml of the SA solution for a hydrogel of SA/AG/Naringin. As detailed in Section 2.2, in the preparation of a Naringin–BG hydrogel, two compositional hydrogels were fully blended. Figure 1 illustrates the production process.

### 2.4. Naringin–BG hydrogel characterization

Naringin–BG hydrogel was confirmed by the scanning electron microscopy (SEM) images and were obtained on a JEM-1400 microscope at an acceleration voltage of 100 kV. The hydrogel scaffolds were freed 12 h and balanced to acquire its dehydrated weights in the assessment of the swelling of the Naringin–BG hydrogel (*W*_d_). Naringin–BG hydrogel were subsequently submerged at room temperature in PBS. Before the water was removed on the samples, samples were deemed to attain wet weights (*W*_s_). The aqueous form samples were measured as (*W*_s_/*W*_d_) and the swelling ratio was recorded.

In order to test the releasing behavior, Naringin was added to a 15 ml tube 1 ml of Naringin–BK and 4 ml simulated bodily fluid (SBF, pH 7.4) previously it was stirred at 37 °C at 100 rpm. In order to test the release behavior Naringin was added to the tube at Naringin–BG. Samples were evaluated with three individual duplicates each at 1, 3, 5, 7, 9, 11, 13 and 21 days. Ultraviolet spectrophotometry detected the absorption of Naringin (at 425 nm). Lastly, the results were analyzed and a chart diagram showing cumulative naringin release was constructed.

### 2.5. Formation of the chondrocyte degenerative

A debilitating chondrocyte models must be established to mimic the inflammatory condition following cartilage defects. In the passage, 3–4 rat chondrocytes were sown in a 6-well platform in a density of 4 × 10^5^ cells. Cells with the medium of 15 ng/ml of IL-1β for one day were incubation after 24 h. The results of the biocompatible material on debilitating chondrocytes were thus simulated and analyzed.

### 2.6. Assessment of the RT-PCR and RNA isolation

The hydrogel of Naringin–BG and BG is distinctly absorbed without serum in cell culture for 3 days. Four times that of hydrogel the size of the culture medium. The Naringin–BG hydrogel extraction and the BG hydrogels extract was obtained with a disposable needle filter of 0.22 μm. Very chondrocyte was split into the three sections (Section I; control, Section II; BG hydrogel, and Section III; Naringin–BG hydrogel). Rather chondrocyte with control medium, hydrogel BG extracts and Naringin–BG hydrogels extracts were cultivated. In addition, the two media added 15% FBS. The mRNA overexpression of ACAN, SOX9, COL2A1 and has been tested in chondrocyte to determine the phenotype of the rat chondrocytes. The degenerative rat chondrocytes were also tested to determine the anti-inflammatory effect of Naringin–BG hydrogels with RT-PCRs in mRNAs concentrations of MMP1, MMP13 and iNOS. The RAW 264.7 cell RAW 264.7 phenotype tested with RAW264.7 cells is evaluated with mRNA ratio of markers M1 and M2 in real-time PCR cell.

For the separation and extraction of RNA, TRIZOL was used. The Pride Script Reverse Transcriptase was synthesized by cDNA, and a Genius SYBR Green Q PCR Master Mix was used to carry out RT-PCR. A housekeeping β-actin gene has been measured for normalization of the expression levels of the genes. A 2-to-t approach to design premiums evaluates the relative genes, the tech Primer 5.0 (Invitrogen Inc., USA). To quantify the average value, each experiment was carried out on three different specimens.

### 2.7. Monitoring the chondrocytes

The chondrocytes are condensed, tallied, and plant in a 6-wave density platform of approximately 4 × 10^4^ cells per each well and 250 μL of suspensions of cell were sucked through a 4-well glass, which is a type of the glass cassettes. In the incubator, the cells were grown (37 °C/5% CO_2_) over 48 h. In the cellular slide glass, the culture medium was removed, and the slide was cleaned with PBS for 3–3 min. The 4% paraformaldehyde solvent was then applied to 200 μL for 30 min at RT and washed with PBS for 3 min at room temperature. The cell dip glass was then used with 0.1% Triton, held at room temperature for 20 min and then washed away. The slide has been removed and blocked for 50 min at room temperature, 10% goat serum (500 μL) was applied to the each slide. Excess liquid was then collected, and the cell diagram glass was diluted with PBS diluting 500 μL of primary collagen type II rabbit anti-rat of 200 times with PBS comprising 10% goat serum, placed in a 4 °C wet box. To clear the liquid, the diaphragm washed with PBS. A cell slide glass was applied to a DAPI and washed with PBS for 2 min daily, after 30 s, immediately. Cells were monitored, and photographs were collected under a fluorescence microscope.

### 2.8. Cartilage defects in animal model

SD rats (30 rats for all the group) were separated into the three sections (10 animals): Section I; control, Section II; BG hydrogel, and Section III; Naringin–BG hydrogel. The rats were divided into two groups. Following anesthesia, on the knee joints medial side, a medial linear incision was performed with a height of 1–1, 5 cm. The femoral–patellar ravine was subsequently dislocated side by side. A complete cylindrical cartilage defect with a diameter of 1.6 mm and a profile of 1 mm was developed using a punch of stainless steels. The BG hydrogel and Naringin–BG hydrogels were then implanted with defects. The control group's defects are blank. Finally, each incision sheet was closed by sutures that were absorbed and the cage reset (Mohamed Subarkhan et al., 2016; Mohan et al., 2018; Balaji et al., 2020). The Department of Orthopedics Animal Research Committee, Department of Orthopedic Surgery, the First Affiliated Hospital, Zhejiang University, School of Medicine, Hangzhou 310003, China, authorized all animal procedures and carried them out in compliance with committee guidelines.

### 2.8. Statistical analysis

Each experiment was repeated at least three times. The mean ± standard deviation of all the data was analyzed in the tables and figures. One-way ANOVA was utilized for statistical evaluation. *P* values < .05 were considered statistically significant (*p < .05, **p < .01, and ***p < .001).

## 3. Results and discussion

### 3.1. Confirmation of the Naringin–BG hydrogel

**Figure 2.**
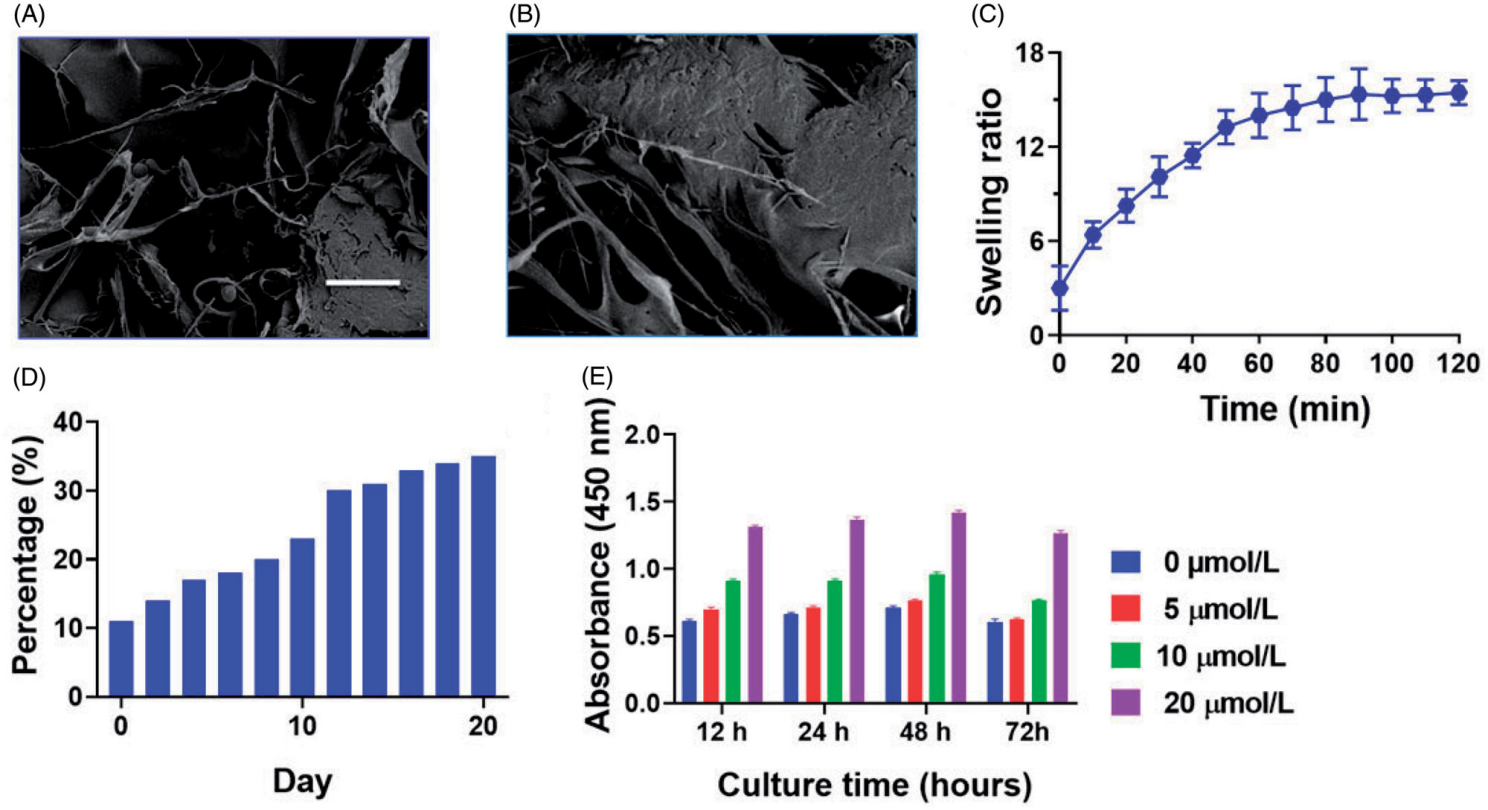
Newly fabricated Naringin–BG hydrogel morphological features were attained by the SEM analysis. A) Naringin–BG hydrogel. Scale bar 5 µm. B) Naringin–BG hydrogel. Scale bar 10 µm. C) Naringin–BG hydrogel swelling ratio assessed by the different time. D) Naringin–BG hydrogel percentage cumulative release. E) Naringin cytocompatibility assessment examined by CCK-8 analysis.

Figure 1 shows a microscopic graphical representation of the Naringin–BG hydrogel gelling process. Three forms of crossed-link, like calcium-ion crossed-link, physical cross-link, and hydrogen crossed links, are primarily used in the gelation process. The interaction between the calcium ion and the slowly released BG calcium ion is triggered by sodium alginate binding. In addition, hydrogen links between alginate and agarose are established as the temperature changes (Liu et al., 2018). In Figure 2(A,B), the SEM picture showing the Naringin–BG hydrogel microstructure after lyophilization. Its key characteristic is a porous system representing cell growth, nutrient exchange and metabolites waste excretion with the outside world. Numerous bioactive glass fragments fitted on the hydrogel's pores. Figure 2(C) reveals that it took approximately 120 min to achieve equilibrium swelling of the Naringin–BG hydrogel samples in PBS solutions. The last swelling capacity for the Naringin–BG hydrogels lyophilized was 15. A first compound explosion of approximately 10% was shown in Figure 2(D). The hydrogel of Naringin–BG shows Figure 2(E) showed that three consecutive days of observation showed that Naringin stimulated chondrocyte proliferation with a concentration of 10 μM. In contrast, 5 μM Naringin did not have a clear impact, and 20 μM had a proliferation inhibition at 48 h. We use 30 times the efficient Naringin concentration in preparing Naringin–BG hydrogel to confirm the substance has adequate drug concentrations when added.

### 3.2. Outcome of the Naringin–BG hydrogels

**Figure 3.**
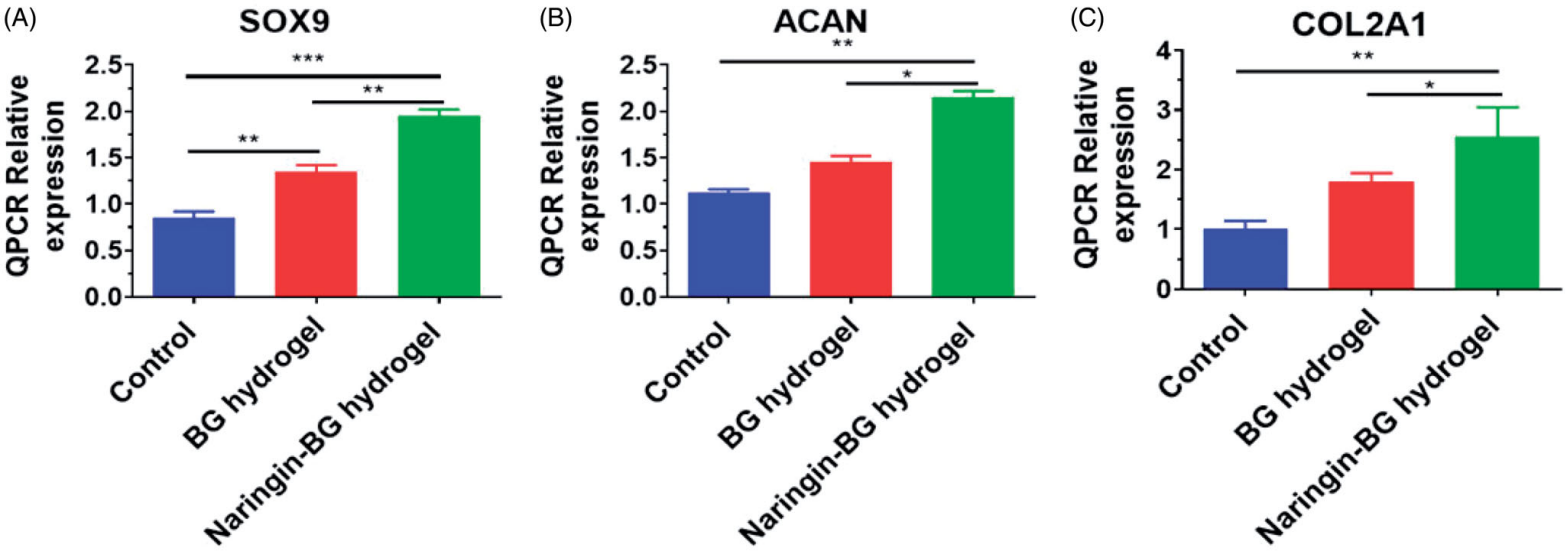
(A–C) Expression level of functional chondrocyte genes (COL2A1, ACAN and SOX9) of the control, BG hydrogels and Naringin–BG hydrogels groups. *, ** and *** denote *p* < .05, *p* < .01 and *p* < .001, respectively.

The preservation of SD rat’s chondrocytes is very significant since they are the only ECM forming cell in cartilage. To investigate the impact of material on the cellular phenotype, the level of expression of the cartilage ECM synthesis (SOx9, COL2A1, ACAN) gene was evaluated in rat chondrocytes grown with BG Hydrogels extracts and Naringin–BG Hydrogels extracts (Figure 3(A–C)). After 48 h, SOX9 expressions in the hydrogel group was 1.5 times higher than in the control class, while in the hydrogel group of Naringin–BG, it was 2.5 times higher (Figure 3(A)). In the hydrogel soaking solution, the relative gene expressions of ACAN were 2.0 times higher than in the control groups after 48 h (Figure 3(B)). In the Naringin**–**BG hydrogels solution, the gene expressions of COL2A1 were higher but not significantly statistic (Figure 3(C)).

**Figure 4.**
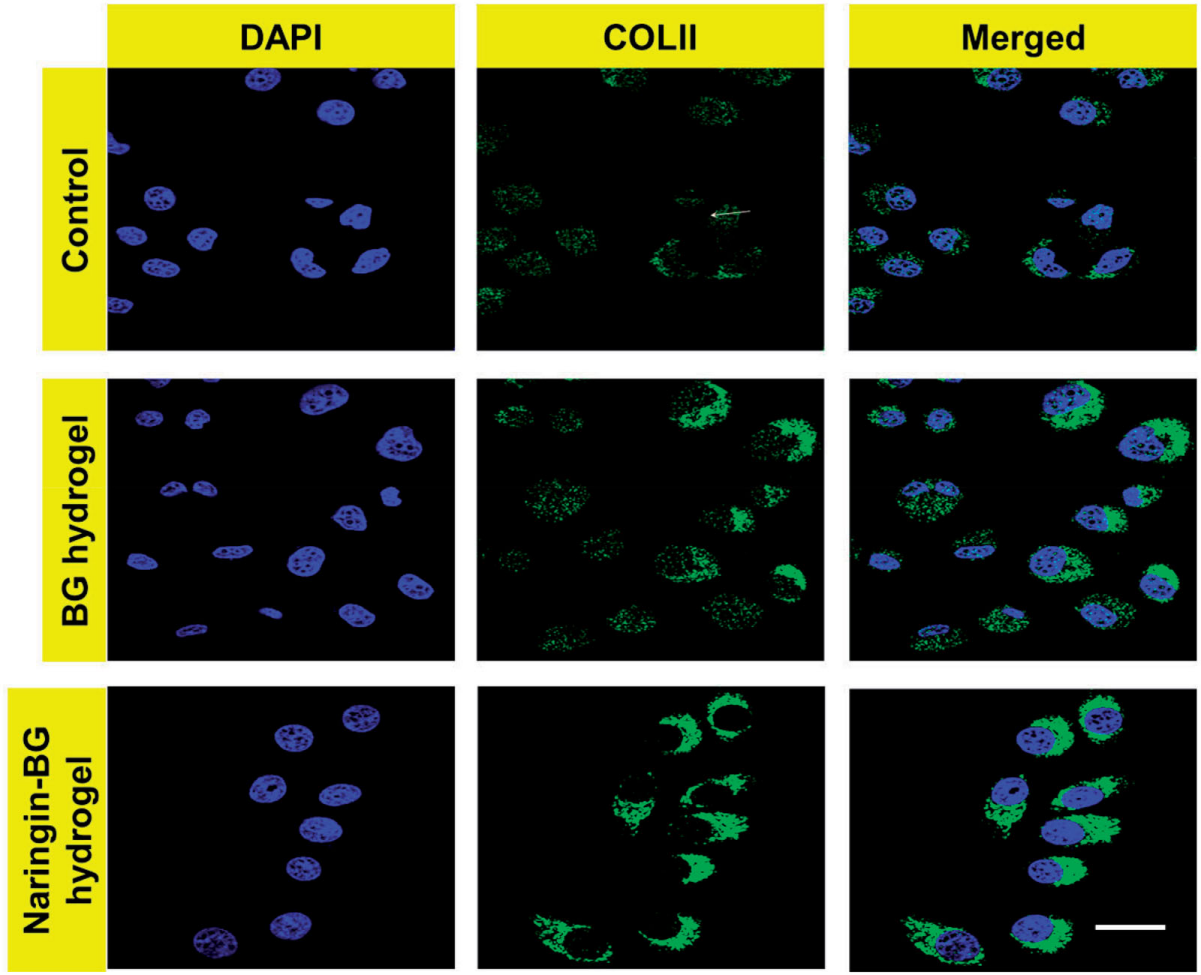
Collagen II of chondrocytes observation by immunofluorescence staining with control, BG hydrogels and Naringin–BG hydrogels groups. Scale bar 10 µm.

Fluorescence microscopy revealed a bleached nucleus, while an ECM showed clear green fluorescence around the cells (Figure 4). Green fluorescence reproduces the collagen type II expression in the ECM about chondrocytes straight away. There were no substantial modifications to cell morphology representing the material's biocompatibility, associated with the untreated groups, the BG hydrogels groups, and the Naringin–BG hydrogels groups. The Naringin–BG hydrogel group's fluorescence strength was largest from both groups, showing that the ECMs in this group are the most excellent collagen type II. The hydrogel Naringin–BG can promote collagen synthesis in rat chondrocytes, defensive the cartilage. These findings show that hydrogel Naringin–BG can hold chondrocytes in a phenotype that produces a matrix.

### 3.3. Results of phenotype of macrophage

**Figure 5.**
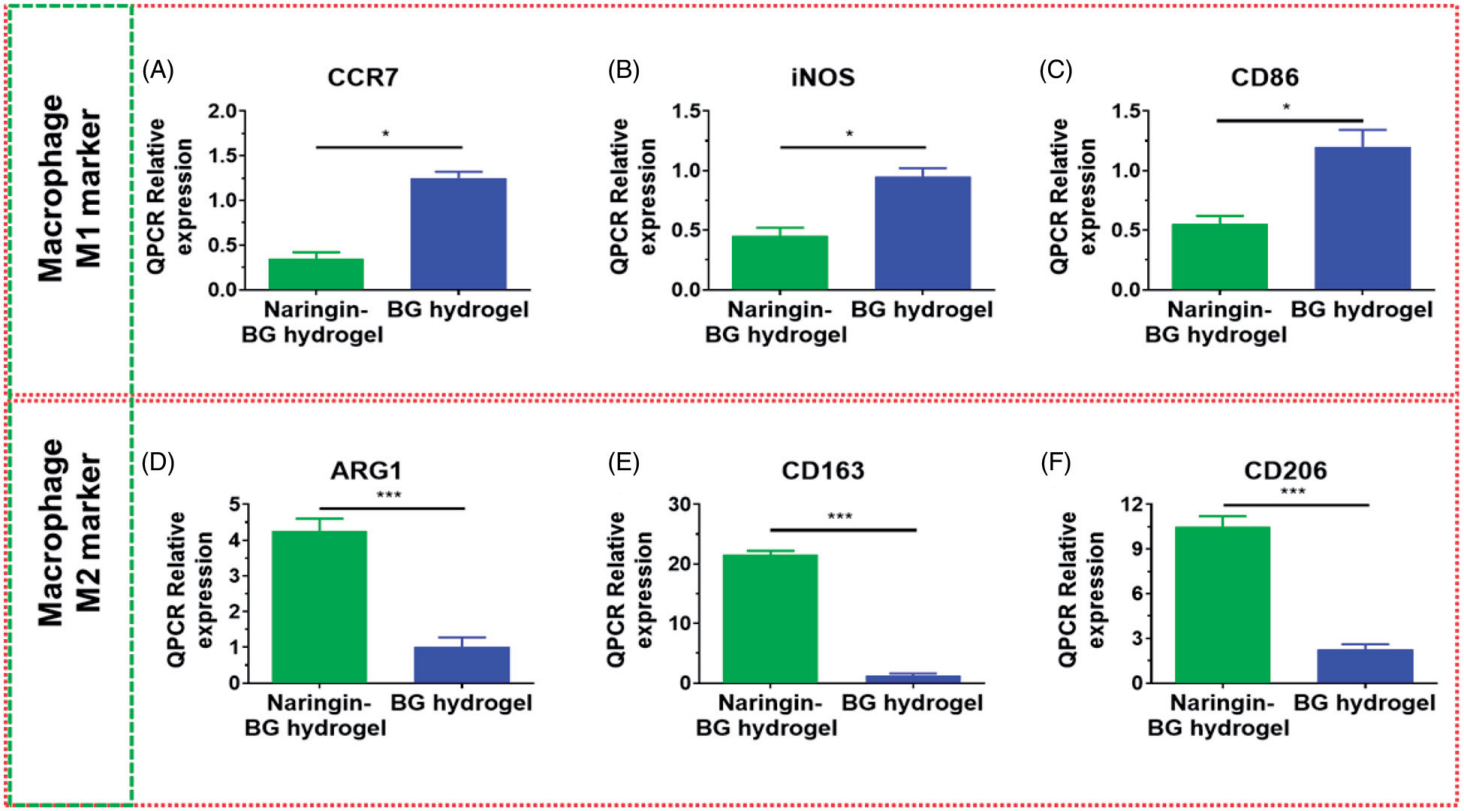
Expression levels of the various genes for BG hydrogel and Naringin–BG hydrogel. (A–C) In RAW264.7 cells macrophage M1 marker genes expression levels for CD86, iNOS and CCR7. (D–F) In RAW264.7 cells macrophage M2 marker genes expression levels for CD206, CD163 and ARG1. * and ** denote *p* < .05, and *p* < .01, respectively.

Phenotype analysis of the target genes of M1 markers and M2 marker for RAW 264.7 cultivated in the BG hydrogels extracts and Naringin–BG hydrogels removal was conducted (Figure 5). After 48 h, the expressions levels for CCR7 were 0.3 times the expressions levels for BG hydrogel groups Naringin–BG (Figure 5(A)). In the Naringin–BG group, iNOS had a relative degree of gene expression approximately 0.5-fold as in the BG hydrogels group (Figure 5(B)). CD86 expressions levels were about 0.7-fold those of the BG hydrogel group. In the Naringin–BG group hydrogels (Figure 5(C)). The M1 expressions level were much lesser in the Naringin–BG hydrogels groups than in the BG hydrogels groups. Hydrogel group of Naringin–BG, the ratio of the expression for ARG1 was 5 to 8-fold in the hydrogels group of BG (Figure 5(D)). The relative level of CD163 gene expressions of the hydrogels groups in Naringin–BG was around 20.1 fold that of the BG hydrogel group (Figure 5(E)). The levels of expressions of CD206 were around 6.3 times that of the hydrogels groups Naringin–BG (Figure 5(F)). In the Naringin–BG hydrogels groups, M2 marker expressions were substantially higher. These findings showed that macrophages to M2 could be activated in the Naringin–BG hydrogel.

### 3.4. Outcomes of chondrocytes expressions

**Figure 6.**
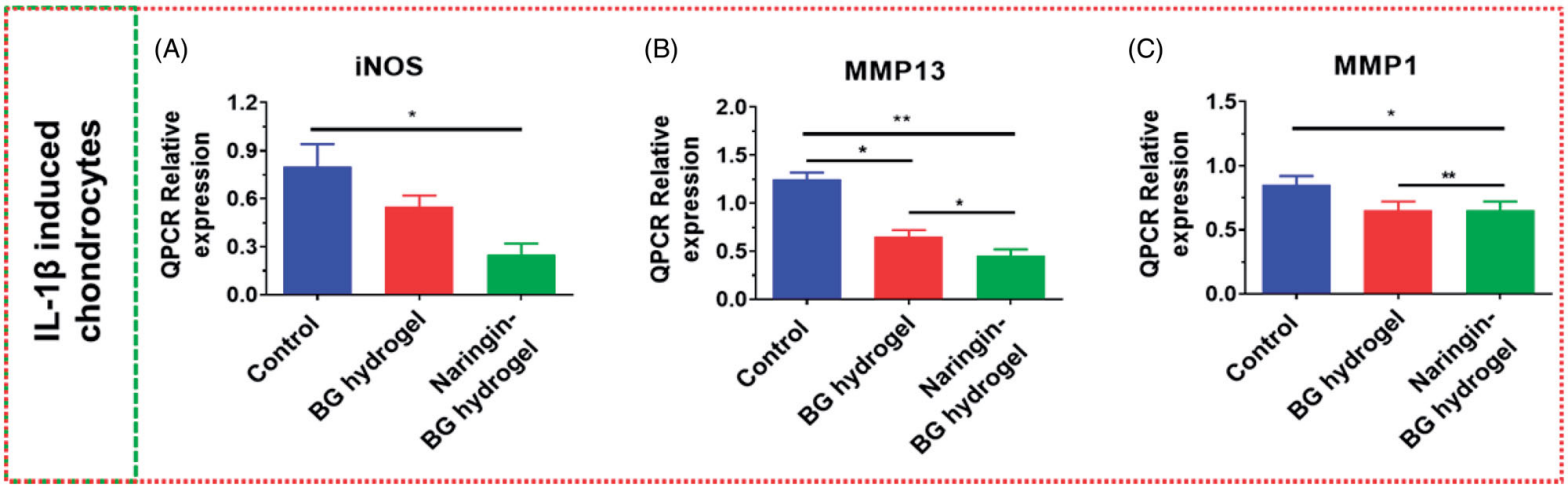
(A–C) Chondrocytes expressions level of inflammatory related genes MMP1 MMP13 and iNOS by IL–1β exposure. * and ** denote *p* < .05, and *p* < .01, respectively.

The expressions ratio in IL-1, β-induced rat chondrocytes, was examined of ECM degradations genes, such as MMP-1, iNOS, and MMP-13. In the BG hydrogel population, iNOS relativity was 48 h above the test group at 0.7 times, compared to Naringin–BG hydrogels groups. The relative expressions of genes were only 0.4-fold the check groups at 48 h (Figure 6(A)). MMP-13 expression levels were approximately 0.6-fold those of the control groups in the hydrogel group, and expression levels were approximately 0.4-fold as much of the control group of Naringin–BG hydrogel (Figure 6(B)). Statistically, the disparity was significant. In the BG hydrogel population, MMP-1 was about 1.0-fold the expression level in the control groups, ∼0.9-fold the levels in the Naringin–BG hydrogels groups (Figure 6(C)). These findings have shown that the hydrogel Naringin–BG can significantly reduce MMP expression, particularly the MMP-13 expression, showing that the hydrogel Naringin–BG can inhibit the ECM degradations and exercise anti-inflammatory properties.

### 3.5. Cartilage repair in animal model

#### 3.5.1. Cartilage defect in animal model

**Figure 7.**
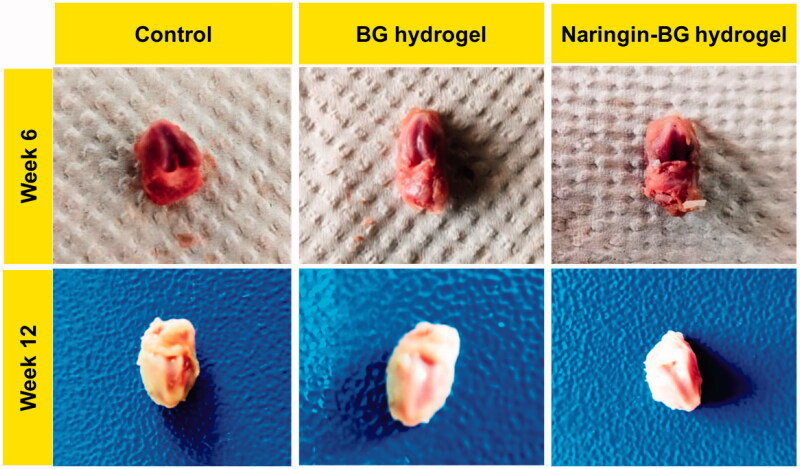
Photograph of cartilage repair in animal model knee joint cavity in SD rats after articular injection of both BG and Naringin–BG.

No swellings, inflammations, and synovial hyperplasias are detected through the uncultivated analysis of the knee joint at both 6 and 12 weeks (Chen et al., 2018; Yi et al., 2019; Aerden et al., 2020). An apparent defect was found 6 weeks after the procedure in the control group, with a definite border across the cartilage defect. There was too little tissue in the control groups associated with the BG hydrogels groups and Naringin–BG groups. The hydrogel groups of both BG and Naringin–BG detected repair tissue with incomplete distribution, limited interaction with nearby tissues, and partial substrate degradations. In week 12, the flaws of the test group were primarily cracked (Figure 7).

**Figure 8.**
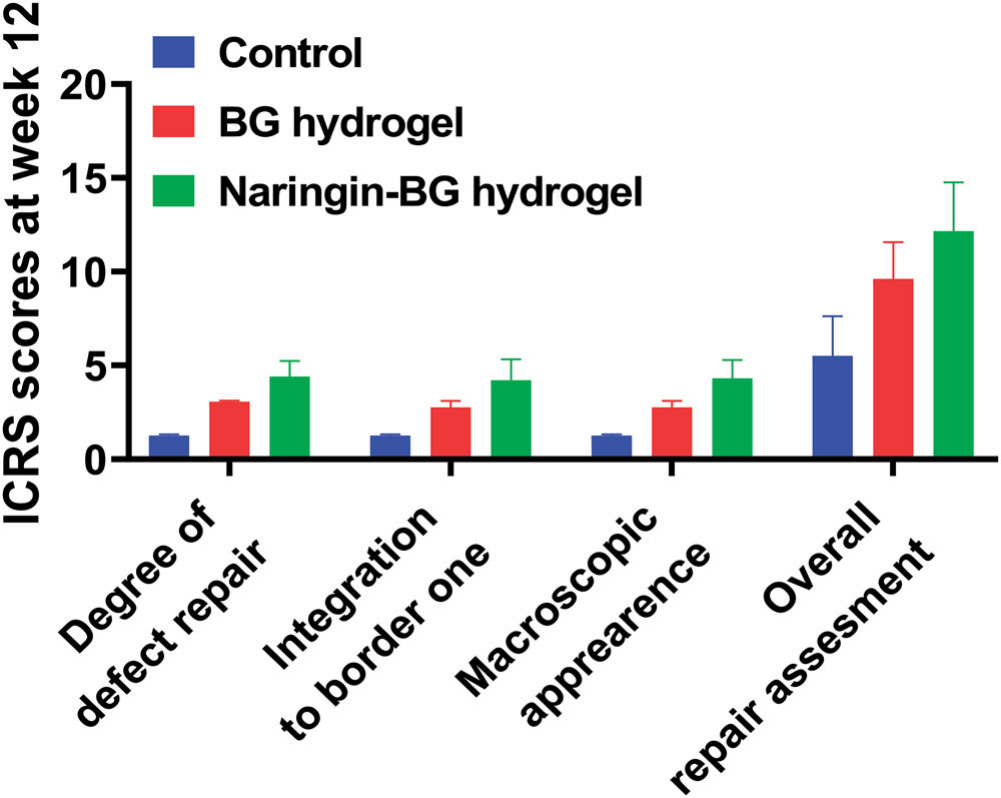
After 12 weeks ICRS surgery ratings for damaged tissues in the control group, BG hydrogel group, and Naringin–BG hydrogel group.

On the other hand, in the BG hydrogel category, there were minor cracks. In the Naringin–BG hydrogel groups, the repair was the greatest apparent of the three groups. The defects were replaced with newer tissues close to normal cartilage in the Naringin–BG hydrogels groups. The new tissues have been combined directly with the underlying tissue (Figure 8). Furthermore, the hydrogel Naringin–BG was well degraded and substituted by the fresh tissues. ICRS ratings showed the consistency of repaired tissue. In the Naringin BG hydrogels groups, the total repair score was slightly higher than that of the other two groups at 12 weeks.

#### 3.5.2. Histopathological model of cartilage repairs

**Figure 9.**
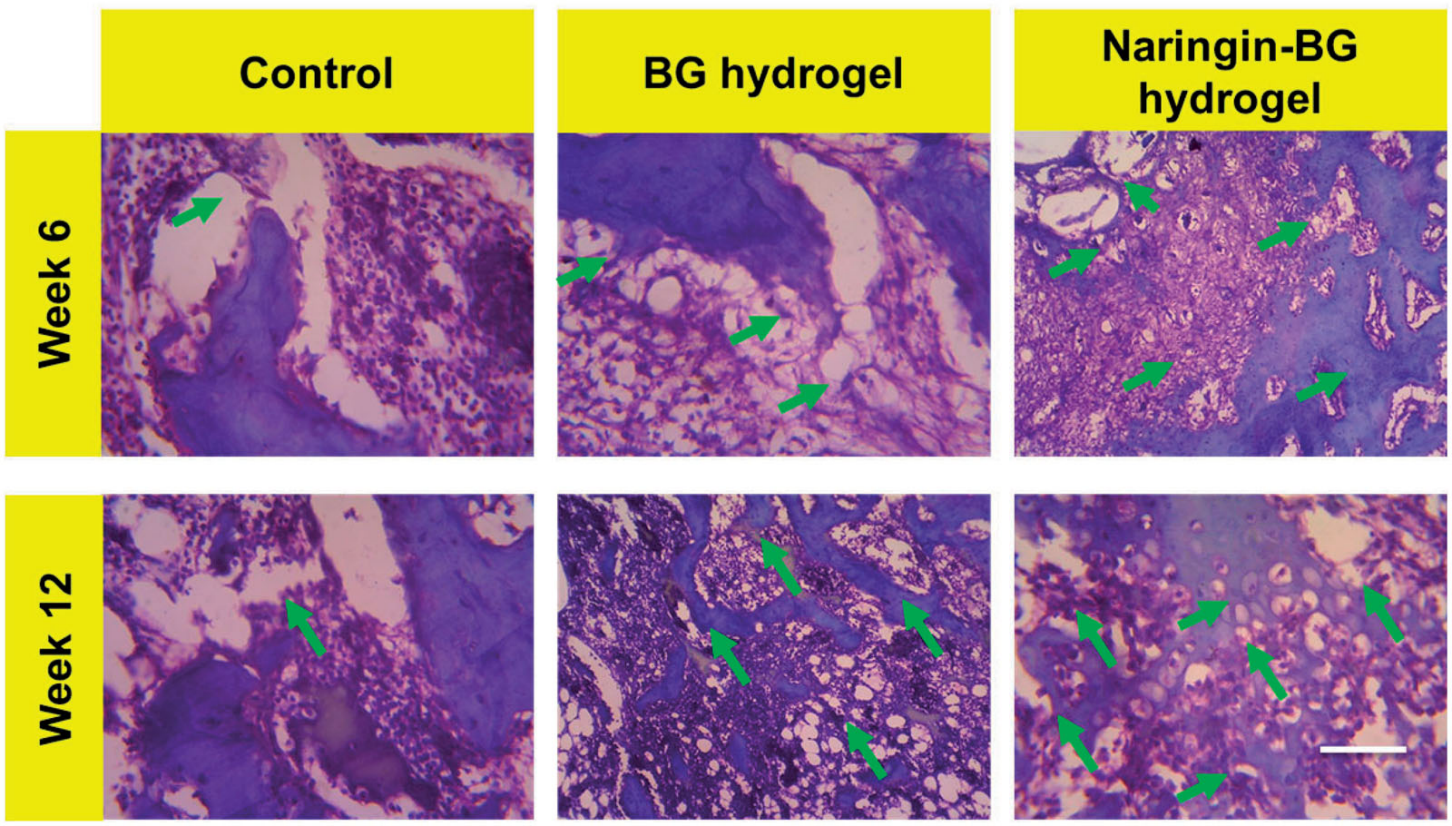
H&E-stained damaged tissue sections from 6 and 12 weeks surgery after treatment with control, BG hydrogel, and Naringin–BG hydrogel groups. Scale bar 100 µm.

Figure 9 shows images of H&E histological staining. A small amount of substance residue was noted after surgery at 6 weeks in BG hydrogels and Naringin–BG hydrogel classes. A fibrous tissue mixture was partially replanted for the defect. Still, the level of the defect repair was considerably better for the Naringin–BG hydrogel group than for the hydrogel BG and control groups. The defects were partly reported. The cartilage and subchondral bone of the new tissue had a distorted border. The newly developed ECM was noticeable on the defect margin, and the cartilage lacuna formation, identical to that displayed in normal tissue, could be observed. In the BG hydrogels community at the 12th week, newly formed tissues mainly consisted of tissues with fibrocartilage and cartilage fabric and were higher than the fault normal tissues. The residuals contents were tainted entirely, and the amount of inflammation cells can be significantly decreased. The border of defects with the corresponding normal tissues almost vanished in the hydrogel of the Naringin–BG group. The more muscular redeveloped cartilage tissues and subchondral bones completed both defects. In the regenerated cartilage can be found the structure rat chondrocytes inside with cartilages lacuna.

**Figure 10.**
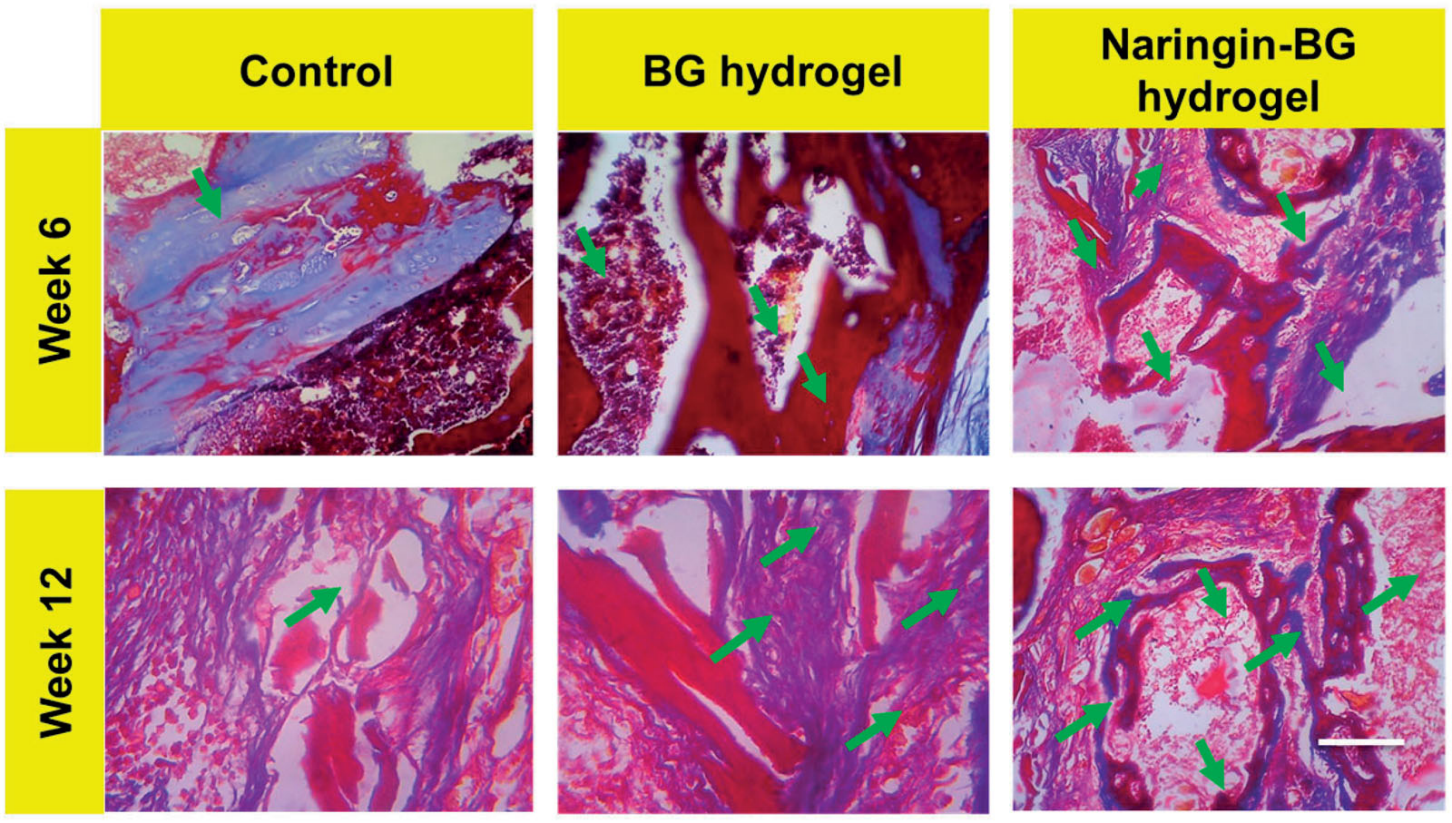
MTS-stained damaged tissue sections from 6 and 12 weeks surgery after treatment with control, BG hydrogel, and Naringin–BG hydrogel groups. Scale bar 100 µm.

The models of the articular cartilage are more distinctive than the H&E stained with saffron oil dye, and Figure 10 shows the findings. In the usual tissue parts staining findings, the high layer was a red, smooth, hyaline cartilage and a blue-green subsoil bone with several moderately sized trabecular structures. The lower layer was blue-green sub-chondral, distributed. There were medium vacuums in the bones, and the transitions from the greater to the minor layers were normal. We saw a distinct color difference in the test group 6 weeks after surgery among the newly healed tissues and normal tissues. The tissues caused by natural cartilage defect repair, similar to the HE staining outcomes, was irregular, and the healed thickness of tissue was somewhat smaller than the average thickness of the tissues. The chondrocyte distributions were uneven in the new cartilages, and the usual red cartilage in the ECM was barely noticeable. Trabecular bone distribution was disordered in the subchondral bones. Six weeks after surgery, the substance was not completely degraded; however, the BG hydrogels were red-fed, and cartilage's original tissue structure was perceived. A minimal amount of substance residue was also found in the Naringin–BG group 6 weeks after the surgery. In the hydrogels BG and control group, the degree of defect repair was considerably higher than that. The cartilages and subchondral bones of the fresh tissues had a distorted border. New ECM cartilage was noticeable at the defects margin, and the cartilages with lacuna formation were found identical to that of the normal tissue. The substance in the Naringin–BG hydrogels group’s repair tissue deteriorated at 12 weeks after surgery and eventually replenished. Compared to the existing cartilage surface of the BG hydrogel group and control group, the chondrocytes distribution was more normal, and there was a finer boundary of the fresh tissue and the unique tissues mixture.

## 4. Conclusion

In brief, we have planned and developed injectable Naringin–BG hydrogels, incorporating Naringin and a BG hydrogels to support articular cartilage development and anti-inflammation properties. We have demonstrated that the Naringin–BG hydrogels can retain the usual chondrocyte morphology, encourage macrophage polarization in the M2 types, efficiently inhibit ECM degradations, and restore the defects tissue cartilages. Outcomes of these findings show that the classy anti-inflammatory drug with hydrogel delivery can facilitate the regeneration of cartilage.
